# Supplementary material for: Oceanic Anoxic Event 2 triggered by Kerguelen volcanism
Source: Nat Commun. 2024 Jun 15;15:5124. doi: 10.1038/s41467-024-49032-3 (PMC11180104; doi:10.1038/s41467-024-49032-3)
Supplement: Supplementary file 1 — Supplementary Information [file 41467_2024_49032_MOESM1_ESM.pdf]

Supplementary materials

Hg as a proxy for volcanism

To account for the relationship between organic carbon and Hg (Supplementary Figure 1), both raw Hg concentrations and Hg normalised to TOC are presented. Oxidation of organic matter can lead to relative increased concentrations of Hg and could provide a false indication of episodes of enhanced volcanic activity[1]. At Sites U1513 and U1516, we found no evidence of TOC oxidation in sediments below the low carbonate horizon, with TOC values (0.3–1 %) typical of marine settings, and the preservation of exceptionally high TOC intervals in the lowermost interval of the low carbonate horizons (Fig. 2). There is potential for organic carbon oxidation above the black shales in the earliest Turonian, which is characterised by very low TOC levels ~0.15%, and we have therefore not measured Hg in this interval.

Euxinic conditions can result in the overprinting of Hg concentrations by comparatively better organic matter preservation and higher TOC values[2]. A lack of photic zone euxinia is evidenced by the absence of isorenieratene (Supplementary Fig. 4) and benthic foraminifera assemblages do not suggest anoxic bottom waters below the low carbonate horizon at Site U1516, with the presence of numerous epifaunal taxa (Fig. 6). Anoxic or dysoxic conditions are also suggested to result in reduced Hg preservation potential, rendering our Hg measurements across supposed anoxic periods (i.e. over the TOC spikes in the black shales) as minimum values. This increases our certainty that our data genuinely reflect an increase in environmental Hg concentrations, rather than reflecting post-depositional alteration. There is also no evidence of buried redox fronts from turbidity currents, also shown to produce peaks in Hg, due to a consistent grain size and lack of turbiditic sedimentary features[3]. Finally, Hg is concentrated closer to continents in modern settings[4], however, our sediments come from marine sites which were distal throughout the late Cenomanian and Turonian and so are unlikely to be affected by this phenomena[3]. We also see no correlation between our Hg data and proxies for terrigenous input (K/Al and εNd; Fig. 6). We therefore consider changes in our Hg and Hg/TOC records as primarily influenced by volcanic activity.

Possible identification of OAE 1-d?

At the base of Sites U1516 (540–490 m) and U1513 (295–268 m), there is little variability in Hg values with relatively low Hg concentrations and Hg/TOC values (Fig. 2). The exception is a single point spike in Hg (450 ng/g) and Hg/TOC (530 ppb/wt%) at 505 m in Site U1516, at the speculative OAE 1d horizon, identified through both biostratigraphy and carbon isotope stratigraphy (grey shading, Fig. 2). Whilst the evidence for OAE1-d is inconclusive, otherwise low background levels of Hg and Hg/TOC through the Albian and Cenomanian demonstrate the magnitude of change across the OAE2 horizon, contextualising the severity of the event.

Tables

Supplementary Table 1. Planktic foraminifera and calcareous nannofossil bioevents at Site U1516[5–7]. *Axopodorhabdus biramiculatus* = *Axopodorhabdus albianus* of some authors. FAD = first appearance datum; LAD = last appearance datum; LO = lowest occurrence; HO highest occurrence. Age of the bioevents according to Gradstein *et al.* (2012). Top and bottom revised Core Composite depth below Sea Floor (rCCSF) in meters, directly corresponds to CCSF-M.

**Event**		**Sample Top**	**Top m rCCSF**	**Sample Bottom**	**Bottom m rCCSF**	**Age**
FAD	*Eiffellitus eximius* (s. Verbeek)	U1516C-28R-1, 110–113 cm	446.70	U1516C-28R-3, 105–108 cm	449.33	92.99
FAD	*Quadrum gartneri*	U1516C-30R-2, 95–96 cm	461.68	U1516C-30R-2, 134–137 cm	462.06	93.55
FAD	*Eprolithus moratus*	U1516C-30R-3, 71–75 cm	462.92	U1516C-30R-3, 131–133 cm	463.48	93.73
FAD	*Eprolithus octopetalus*	U1516C-31R-2, 8–11 cm	465.79	U1516C-31R-2, 97–100 cm	466.68	93.80
LAD	*Helenea chiastia*	U1516C-31R-2, 8–11 cm	465.79	U1516C-32R-1, 97–100 cm	470.89	93.90
LAD	*Axopodorhabdus biramiculatus*	U1516C-32R-1, 131–134 cm	471.24	U1516C-32R-2, 4–7 cm	471.48	94.20
LAD	*Lithraphidites acutus*	U1516C-32R-3, 111–113 cm	474.05	U1516C-32R-CC, 1–5 cm	474.12	94.40
LAD	*Gartnerago nanum*	U1516C-33R-5, 3–7 cm	479.29	U1516C-33R-CC, 1–5 cm	480.07	94.79
FAD	*Lithraphidites acutus*	U1516C-34R-4, 6–8 cm	484.29	U1516C-35R-1, 87–90 cm	485.76	96.16

Supplementary Table 2. Planktic foraminifera and calcareous nannofossil bioevents at Site U1513. The same abbreviations are used as in Supplementary Table 1.[7]

Event		Sample Top	Top m rCCSF	Sample Bottom	Bottom m rCCSF	Age
FAD	*Quadrum gartneri*	U1513D-15R-CC, 7–12 cm	227.80	U1513C-41X-CC, 27–30 cm	229.47	93.55
FAD	*Eprolithus moratus*	U1513D-17R-2, 120–123 cm	236.65	U1513C-43X-1, 35–38 cm	237.35	93.73
FAD	*Eprolithus octopetalus*	U1513D-18R-2, 95–98 cm	241.33	U1513D-18R-2, 140–143 cm	241.78	93.79
LAD	*Helenea chiastia*	U1513D-18R-3, 133–136 cm	243.21	U1513C-45X-2, 144–147 cm	247.60	93.90
HO	*Axopodorhabdus biramiculatus*	U1513C-45X-2, 144–147 cm	247.60	U1513C-45X-3, 16–18 cm	247.81	94.20
LAD	*Lithraphidites acutus*	U1513D-20R-1, 37–40 cm	248.97	U1513D-20R-1, 145–148 cm	250.05	94.40
LAD	*Gartnerago nanum/ponticula*	U1513C-47X-1, 140–143 cm	256.65	U1513C-47X-2, 135–138 cm	258.49	94.79
FAD	*Lithraphidites acutus*	U1513C-48X-1, 65–68 cm	264.47	U1513C-48X-2, 25–28 cm	265.52	96.16
FAD	*Gartnerago obliquum/segmentatum*	U1513C-48X-4, 30–33 cm	268.57	U1513D-22R-3, 25–28 cm	271.13	98.26
LAD	*Watznaueria britannica*	U1513C-48X-4, 30–33 cm	268.57	U1513D-22R-3, 25–28 cm	271.13	100.03
LO	*Corollithion kennedyi*	U1513D-22R-3, 25–28 cm	271.13	U1513C-48X-6, 65–68 cm	271.31	100.45

Supplementary Table 3. Benthic foraminiferal palaeoecological designations used to calculate the 'Infauna/high productivity %' plot in Fig. 6. Palaeobathymetric information is also provided. *Gaudryina australis* and *Cibicides complanata* are placed in groups based on their shell $\delta^{13}C$ (Supplementary Fig. 10). *Gavelinella* spp. (apart from *Gavelinella nacatochensis*) are not assigned a group due to uncertain interpretations in the literature.

**Taxon**	**Group**	**Notes**
Bolivina spp.	Infaunal/high prod/low oxygen	Part of 'low oxygen' assemblages, modern and Cretaceous[9]<br>Infaunal, deposit feeders – bacterial/detrital scavengers[10].<br>Middle-outer neritic and upper-middle bathyal. Living in fine-grained calcareous or siliceous mud[11].<br>Recorded as anoxic indicators in Miocene, Pleistocene and Holocene[12].
Bulimina triangularis	Infaunal/high prod/low oxygen	Genus suggested to dominate modern low-oxygen assemblages[9].
Lenticulina spp.	Infaunal/high prod/low oxygen	Indicative of dysoxia[9].<br>Epifaunal/shallow infaunal, active deposit feeder (grazing herbivores/detritivores), lives in fine-grained sands or calcareous or siliceous muds. Neritic to upper-middle bathyal[10].<br>Modern species live epifaunal to shallow infaunal. Opportunistic and morphologically adapted to move vertically in sediment in response to availability of food and oxygen. Tolerant to oxygen depletion. Adapted to a wide variety of environmental conditions. Generally smooth or little ornamented test suitable for rapid burrowing[13].<br>Suggested infaunal based on carbon isotope comparison (this study).
Osangularia spp.	Infaunal/high prod/low oxygen	Opportunistic, benefitting from eutrophic conditions by reproducing at high rates, leading to a dominance in low-diversity assemblages[9].<br>Epifaunal, active deposit feeder. Living in fine-grained sands, or calcareous or siliceous muds. Middle-outer neritic to upper bathyal[10].
Pleurostomella spp.	Infaunal/high prod/low oxygen	Highly tolerant to oxygen deficiency[9].<br>Infaunal deposit feeder. Living in fine-grained calcareous or siliceous muds. Middle-outer neritic to upper-middle bathyal[10].
Praebulimina nannina	Infaunal/high prod/low oxygen	Deep water species[14].<br>Low oxygen tolerant and epifaunal[15].<br>Low oxygen settings[10,16].

Praebulimina spp.	Infaunal/high prod/low oxygen	Infaunal habit[17].<br>Indicates eutrophic and lower oxygenated conditions in other Cenomanian/Turonian sections[18].<br>Infaunal deposit feeder. Living in fine-grained calcareous or siliceous muds. Middle-outer neritic to upper-middle bathyal[10].
Pseudouvigerina cimbrica	Infaunal/high prod/low oxygen	Upper-middle shelf to upper bathyal. Modern genus tolerates high organic carbon[19].
Spiroplectammina spp.	Infaunal/high prod/low oxygen	Infaunal deposit feeder, living in fine-grained calcareous or siliceous muds. Paralic, neritic and bathyal[10].
Tappanina laciniosa	Infaunal/high prod/low oxygen	Infaunal. Typical for highly eutrophic environments, and dysoxic to nearly anoxic facies[18].<br>Demerara Rise: first benthic to repopulate seafloor after anoxic conditions, indicating initiation of oxygenation at seafloor[18].<br>USA Western Interior Seaway: associated with oxygenated to dysoxic conditions prior to most severe part of OAE2[18].
Berthelina spp.	Epifaunal	Considered as epifaunal, living in fine-grained sands or calcareous or siliceous muds. Middle-outer neritic to upper-middle bathyal[10].
Cibicides spp.	Epifaunal	Epifaunal, passive (attached) herbivore. Living in fine-grained sands, or calcareous or siliceous muds. Middle-outer neritic and upper-middle bathyal[10].
Cibicidoides spp.	Epifaunal	Epifaunal, living in fine-grained sands, or calcareous or siliceous muds. Middle-outer neritic to upper-middle bathyal[10].
Gavelinella nacatochensis	Epifaunal	Epifaunal, living in fine-grained calcareous or siliceous muds. Neritic to upper bathyal[10].
Gaudryina australis	Epifaunal	Suggested epifauna based on carbon isotope comparison (this study).
Lingulogavelinella spp.	Epifaunal	Epifaunal, browsing herbivores and active deposit feeders. Living in fine-grained calcareous or siliceous muds. Neritic and upper bathyal[10].
Nuttallinella spp.	Epifaunal	Epifaunal, free-living deposit-feeder, fine-grained sands or calcareous or siliceous muds. Middle-outer neritic to upper-middle bathyal[10].
Patellinella spp.	Epifaunal	Epifaunal, living in bioclastic fine-grained sands or calcareous muds. Algal patch-reefs and neritic[10].

Valvulineria lenticula	Epifaunal	Deep water species[14].<br>Genus is considered as epifaunal, browsing herbivore or active deposit feeder. Living in fine-grained calcareous or siliceous muds. Neritic to upper bathyal[10].
Ammodiscus spp.	Intermediate infauna	Epifaunal to shallow infaunal, active deposit feeder. Lives in fine-grained sands or siliceous muds. Bathyal[10].
Brunsvigella thoerensis	Intermediate infauna	Uncoiled vaginulinids known as epifaunal to shallow infaunal, deposit feeders. Living in fine-grained sand, or calcareous or siliceous muds. Neritic to upper-middle bathyal[10].
Cibicides complanata	Intermediate infauna	Suggested intermediate infauna based on carbon isotope comparison (this study).
Citharina spp.	Intermediate infauna	Epifaunal to shallow infaunal, deposit feeder. Lives in siliceous muds. Outer neritic to upper bathyal[10].
Dentalina spp.	Intermediate infauna	Epifaunal to shallow infaunal, deposit feeder. Lives in fine-grained sands, or calcareous or siliceous mud. Neritic to upper/middle bathyal[10].
Ellipsoidella spp.	Intermediate infauna	Epifaunal or infaunal. Lives in fine-grained or calcareous muds. Neritic and upper/middle bathyal[10].
Frondicularia spp.	Intermediate infauna	Epifaunal to shallow infaunal, deposit feeder. Live in siliceous muds. Outer neritic to upper bathyal[10].
Gaudryina spp.	Intermediate infauna	Modern: shallow infaunal habitat, mesotrophic to eutrophic, oxygenated environments. Cretaceous: epifaunal to shallow infaunal deposit feeders, aerobic but tolerant of somewhat decreased oxygen levels[13].<br>Epifaunal to shallow infaunal, active deposit feeder or passive (attached) herbivore. Living in fine-grained sands, or calcareous or siliceous muds. Middle-outer neritic to upper-middle bathyal[10].
Gyroidinoides spp.	Intermediate infauna	Modern: shallow infaunal habitat, mesotrophic to eutrophic, but still oxygenated environments. Cretaceous: epifaunal to shallow infaunal deposit feeder, aerobic but tolerant of somewhat decreased oxygen levels[13].<br>Epifaunal to shallow infaunal, active deposit feeders or passive (attached) herbivores. Living in fine-grained sands, or calcareous or siliceous muds. Middle-outer neritic to upper-middle bathyal[10].
Gyroidinoides globosus	Intermediate infauna	Common at deep bathyal to abyssal settings[20].

Gyroidinoides lenticulus	Intermediate infauna	Cosmopolitan, upper bathyal[19].
Gyroidinoides primitiva	Intermediate infauna	Outer shelf to upper slope[21].
Gyroidinoides quadratus	Intermediate infauna	Cosmopolitan species[22].<br>Bathyal[23], middle bathyal[24], lower bathyal[17].
Marginulina spp.	Intermediate infauna	Epifaunal to shallow infaunal deposit feeder. Lives in fine-grained sands, or calcareous or siliceous mud. Neritic to upper/middle bathyal[10].
Nodosaria spp.	Intermediate infauna	Epifaunal to shallow infaunal, deposit feeder. Lives in fine-grained sands, or calcareous or siliceous mud. Neritic to upper/middle bathyal[10].
Palaeopolymorphina spp.	Intermediate infauna	Polymorphinids are known as epifaunal and infaunal. Living in fine-grained calcareous or siliceous muds. Neritic to upper/middle bathyal[10].
Planularia spp.	Intermediate infauna	Epifaunal to shallow infaunal, active deposit feeder. Lives in fine-grained sands, or calcareous or siliceous mud. Neritic to upper/middle bathyal[10].
Ramulina spp.	Intermediate infauna	Epifaunal to shallow infaunal, deposit feeder. Lives in fine-grained sands, or calcareous or siliceous mud. Neritic to upper/middle bathyal[10].
Saracenaria spp.	Intermediate infauna	Epifaunal to shallow infaunal, deposit feeder. Lives in fine-grained sands, or calcareous or siliceous mud. Neritic to upper/middle bathyal[10].

Figures

Supplementary Figure 1 – Map of Site Locations

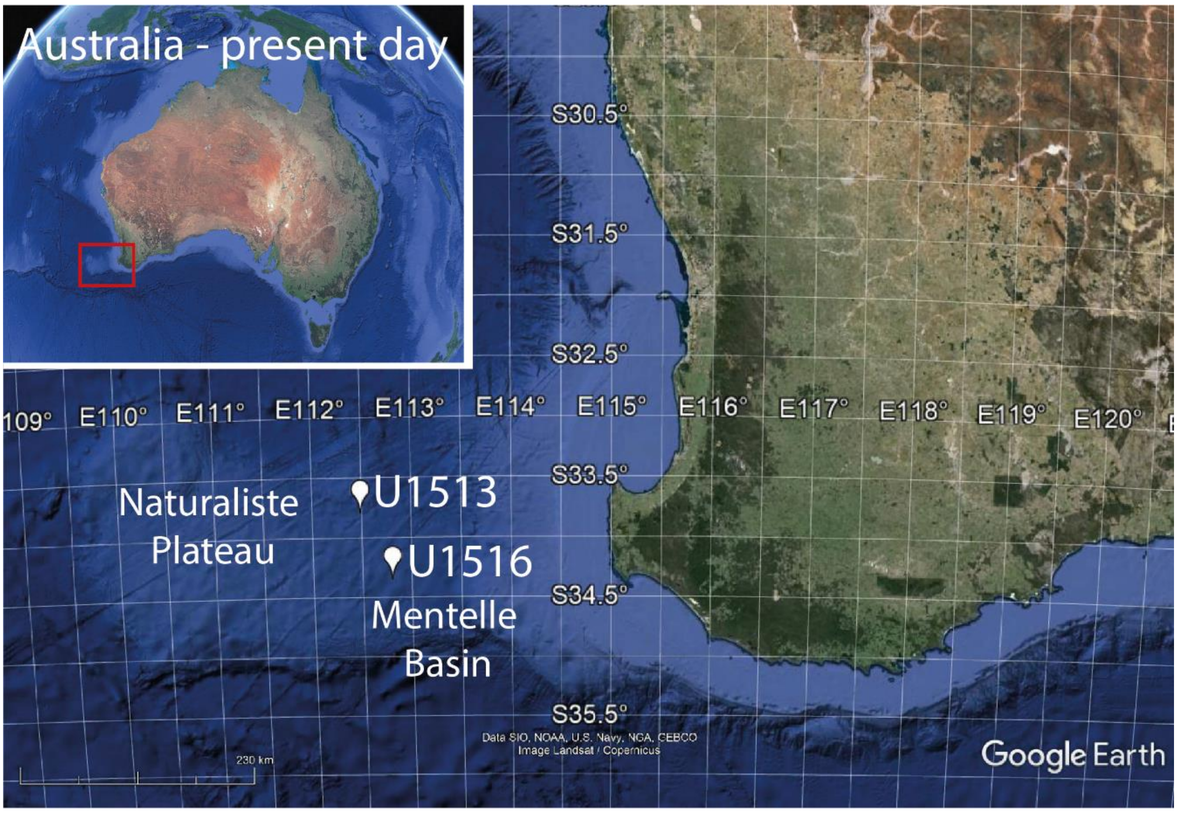

Map of Site Locations. Present day map of SW Australia showing location of IODP Sites U1513 and U1516. Created using Google Earth Pro V7.3.4.8248 (January 17, 2023), SW Australia, Data: SIO, NOAA, U.S. Navy, NGA, GEBCO, Image Landsat / Copernicus. Site locations from Huber et al., (2019)[3].

Supplementary Figure 2 – Creation of composite benthic foraminiferal δ^{13}C curve

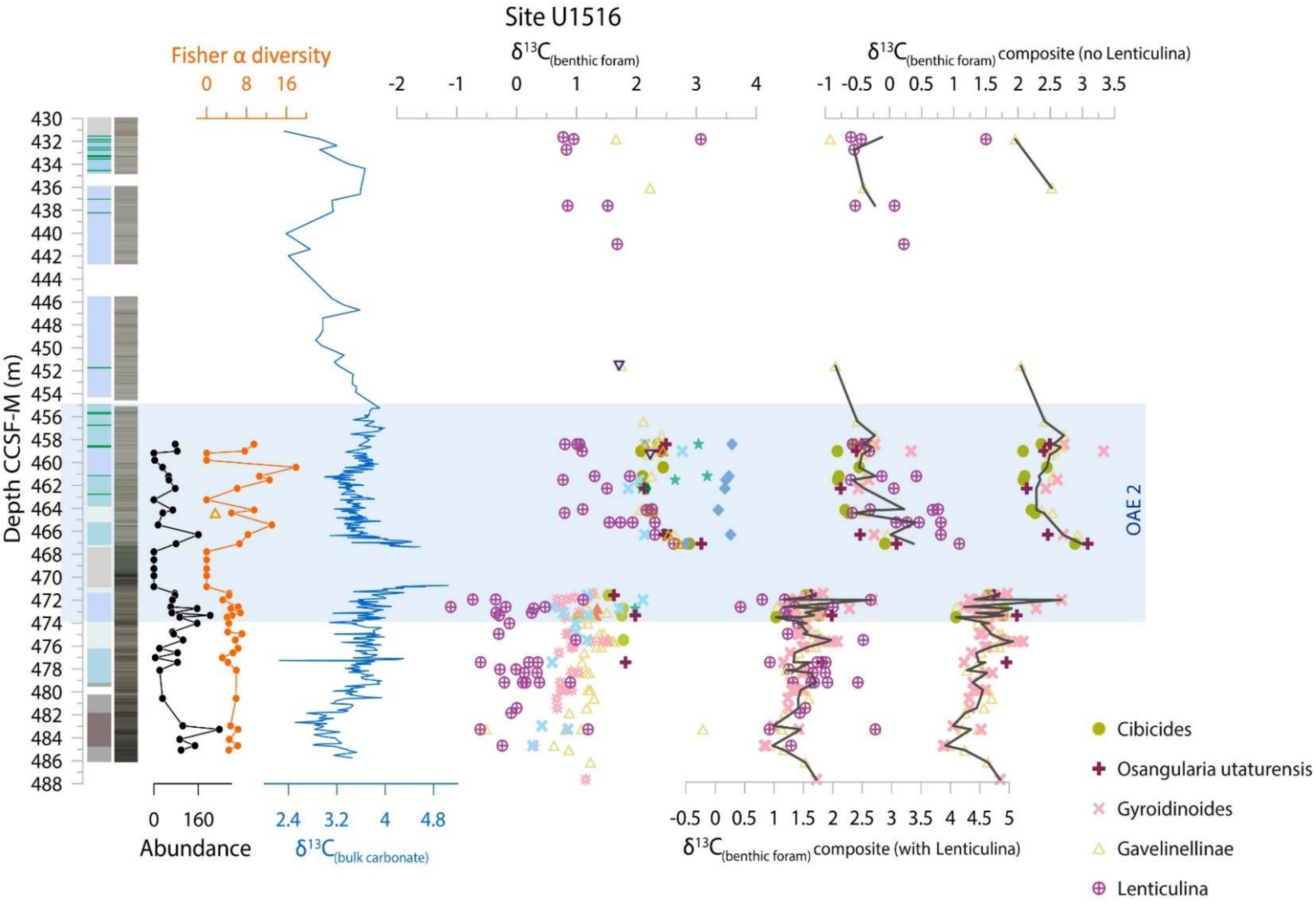

Creation of composite benthic foraminiferal δ^{13}C curve. δ^{13}C data for benthic foraminifera from this study and from Petrizzo et al. (2021), plotted against abundance of individuals, Fisher α Diversity and bulk stable isotope data. Blue shaded area is Ocean Anoxic Event 2. Composite curves are plotted with (left) and without (right) *Lenticulina*, which is a diverse group and may represent a variety of species which do not have a consistent offset from the seawater signal. However, both composite curves demonstrate prolonged changes to the assemblage after the low carbonate horizon.

Supplementary Figure 3 - Creation of composite benthic foraminiferal δ^{18}O curve

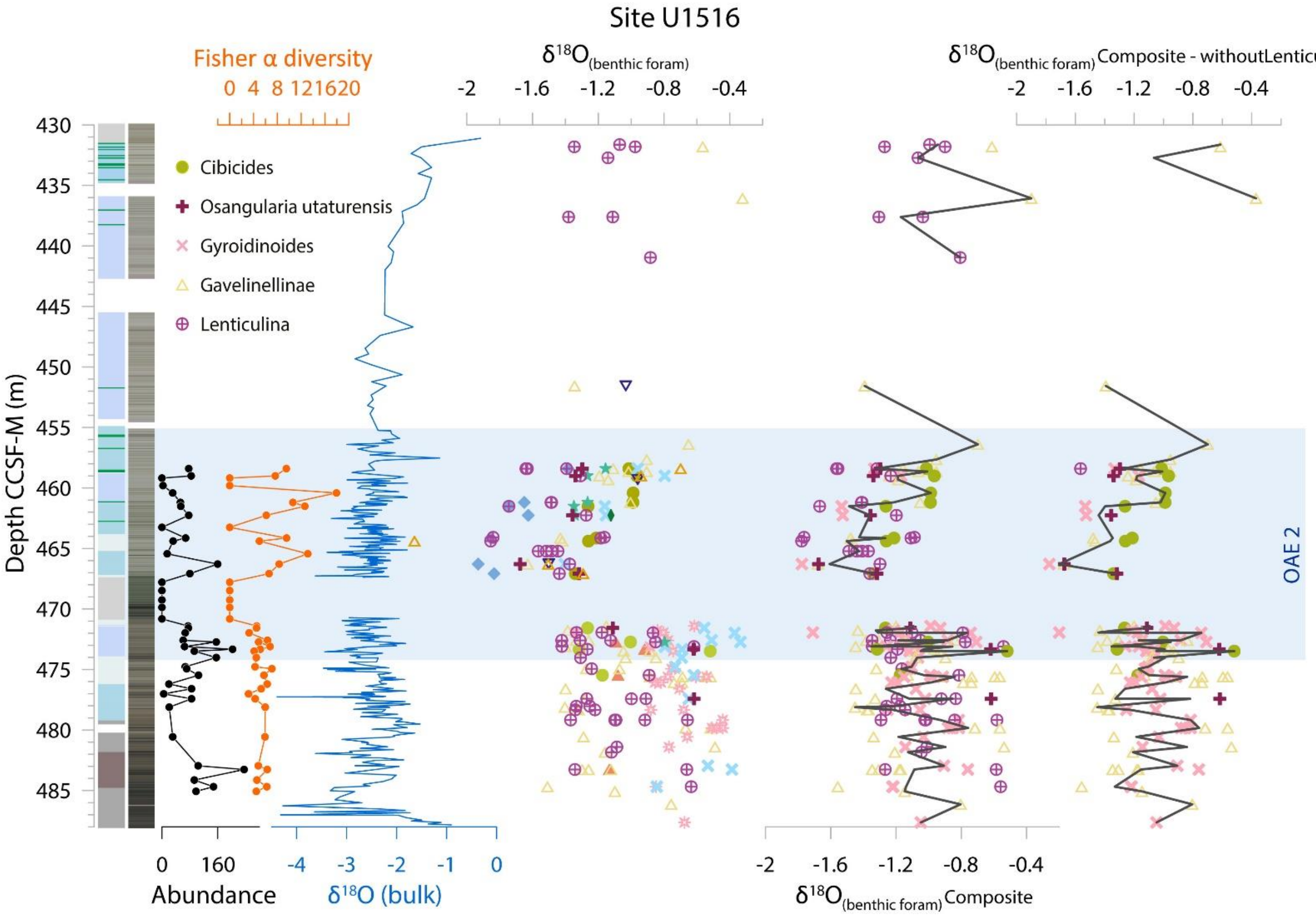

Creation of composite benthic foraminiferal δ^{18}O curve. δ^{18}O data for benthic foraminifera from this study and from Petrizzo et al. (2021), plotted against abundance of individuals, Fisher α Diversity and bulk stable isotope data. Blue shaded area is Ocean Anoxic Event 2. Composite curves are plotted with (left) and without (right) *Lenticulina*, which is a diverse group and may represent a variety of species which do not have a consistent offset from the seawater signal.

Supplementary Figure 4 – Weak correlation between Hg and terrigenous input proxies

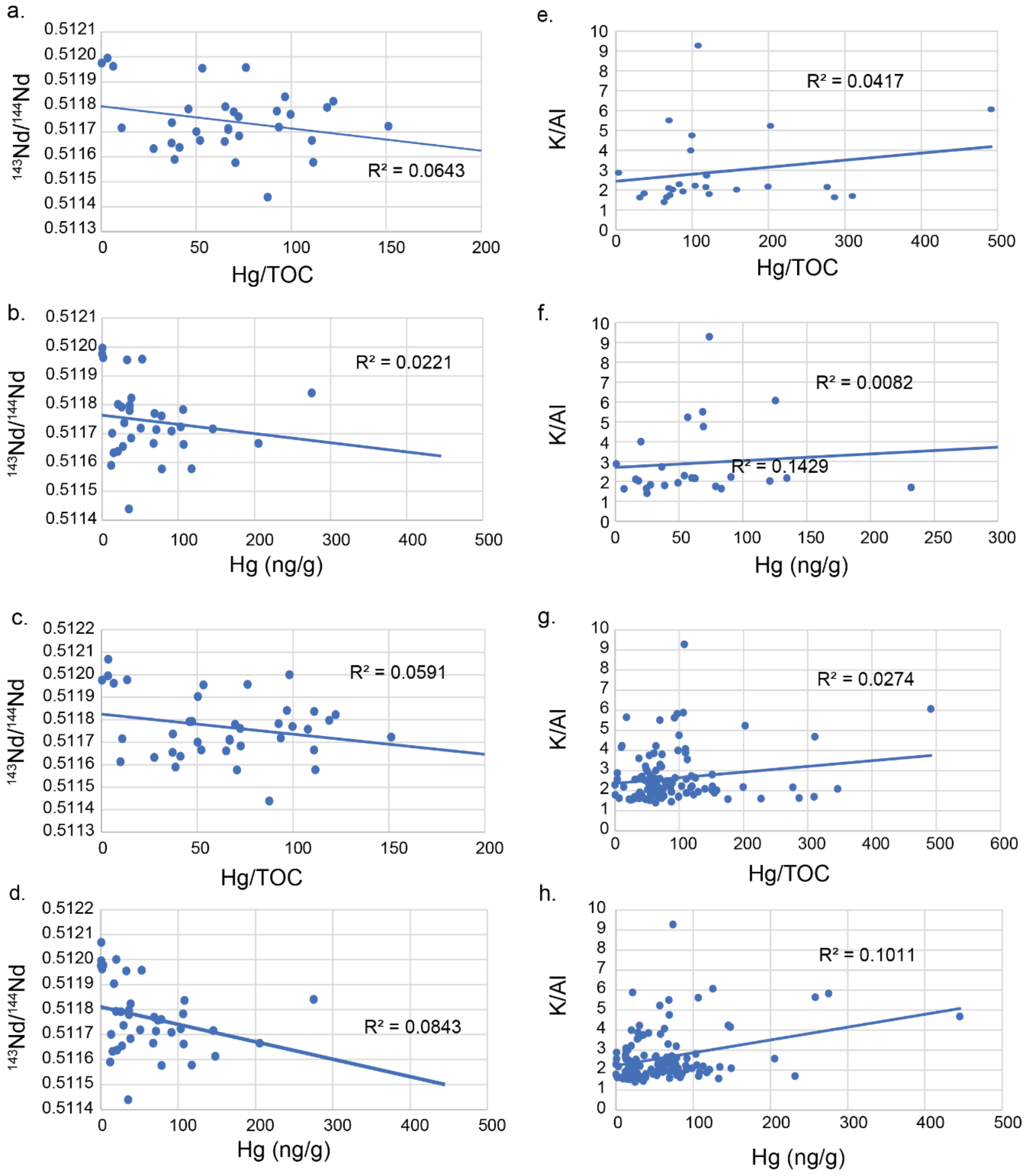

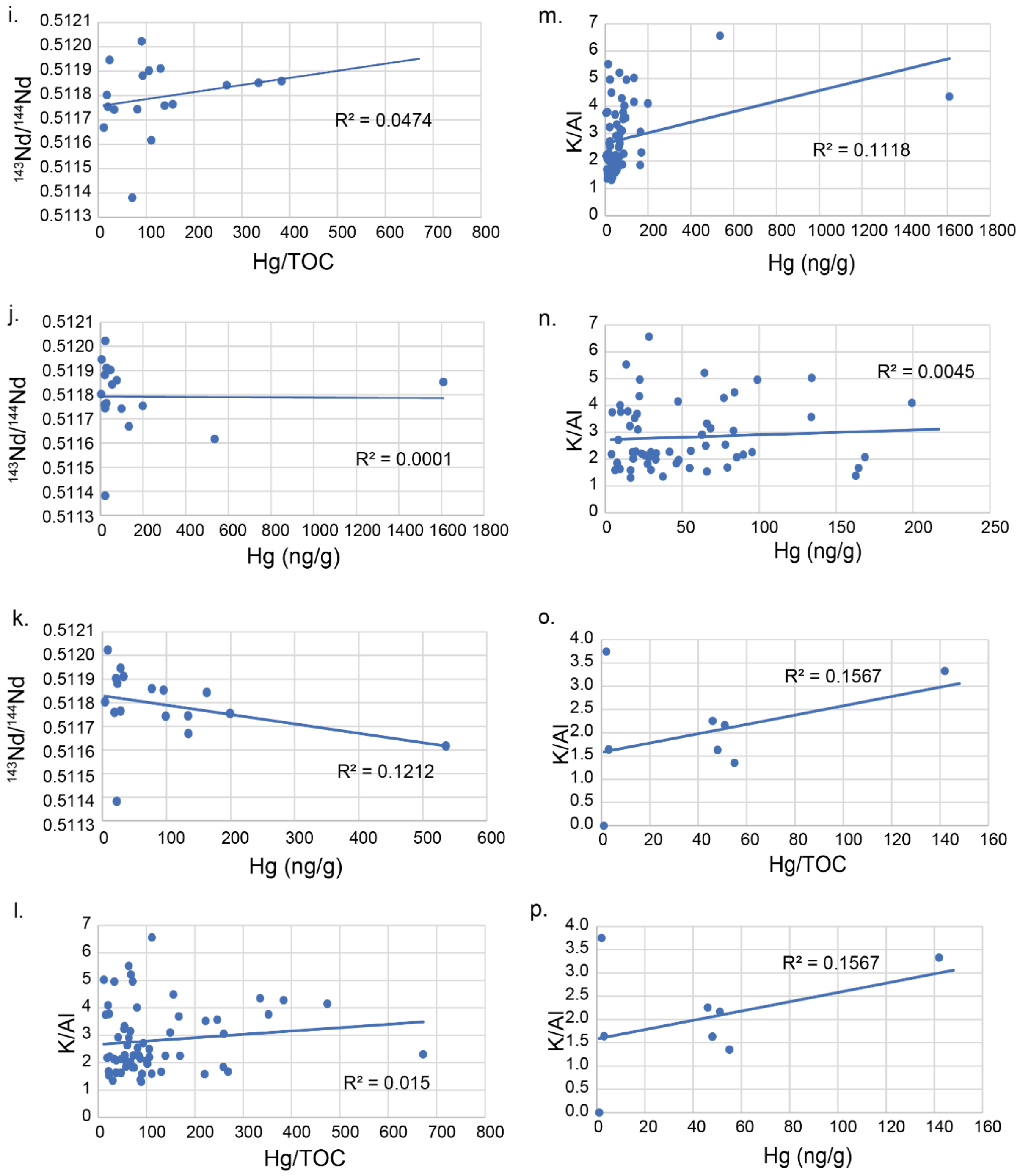

Weak correlation between mercury and terrigenous input proxies. Cross plots of Hg and Hg/Total Organic Carbon (TOC) against proxies for weathering – K/Al and ^{143}Nd/^{144}Nd at sites U1513 (a – h) and U1516 (i – p). These show a lack of correlation between Hg and proxies for terrigenous input, indicating our Hg values are not primarily influenced by transported terrignous material, common in marginal settings. Linear trend strength is displayed in the R^2 value on each graph. XRF data were collected separately to Hg data, and not all Hg samples have a directly corresponding sample with XRF values available. As such, two sets of graphs are figured; the first 'exact sample depth match' shows only data for which XRF data and Hg data are available from within the same sample. The second, 'approximate sample depth match' shows all Hg data points, matched to the next nearest XRF sample (usually <1cm away in the sediment core).

U1513 (a – h): a) $^{143}Nd/^{144}Nd$ plotted against Hg/TOC - exact sample depth match. $R^2 = 0.0147$; b) $^{143}Nd/^{144}Nd$ plotted against Hg - exact sample depth match. $R^2 = 0.1429$; c) $^{143}Nd/^{144}Nd$ plotted against Hg/TOC - approximate sample depth match. $R^2 = 0.0921$; d) $^{143}Nd/^{144}Nd$ plotted against Hg - approximate sample depth match. $R^2 = 0.3318$; e) K/Al plotted against H/TOC – exact sample depth match. $R^2 = 0.0417$; f) K/Al plotted against Hg – exact sample depth match. $R^2 = 0.0082$; g) K/Al plotted against Hg/TOC – apprximate sample depth match. $R^2 = 0.0274$; h) K/Al plotted against Hg– apprximate sample depth match. $R^2 = 0.1011$.
U1516 (i – p): i) $^{143}Nd/^{144}Nd$ plotted against Hg/TOC - approximate sample depth match. $R^2 = 0.0474$; j) $^{143}Nd/^{144}Nd$ plotted against Hg - approximate sample depth match. $R^2 = 0.0001$; k) $^{143}Nd/^{144}Nd$ plotted against Hg/TOC - approximate sample depth match, Hg < 600 ng/g. $R^2 = 0.1212$; l) K/Al plotted against Hg - approximate sample depth match. $R^2 = 0.015$; m) K/Al plotted against H/TOC – approximate sample depth match. $R^2 = 0.1118$; n) K/Al plotted against Hg – approximate sample depth match, Hg < 600 ng/g. $R^2 = 0.0045$; o) K/Al plotted against Hg/TOC – exact sample depth match. $R^2 = 0.1567$; p) K/Al plotted against Hg– exact sample depth match. $R^2 = 0.1567$.

Supplementary Figure 5 – Absence of isorenieratine

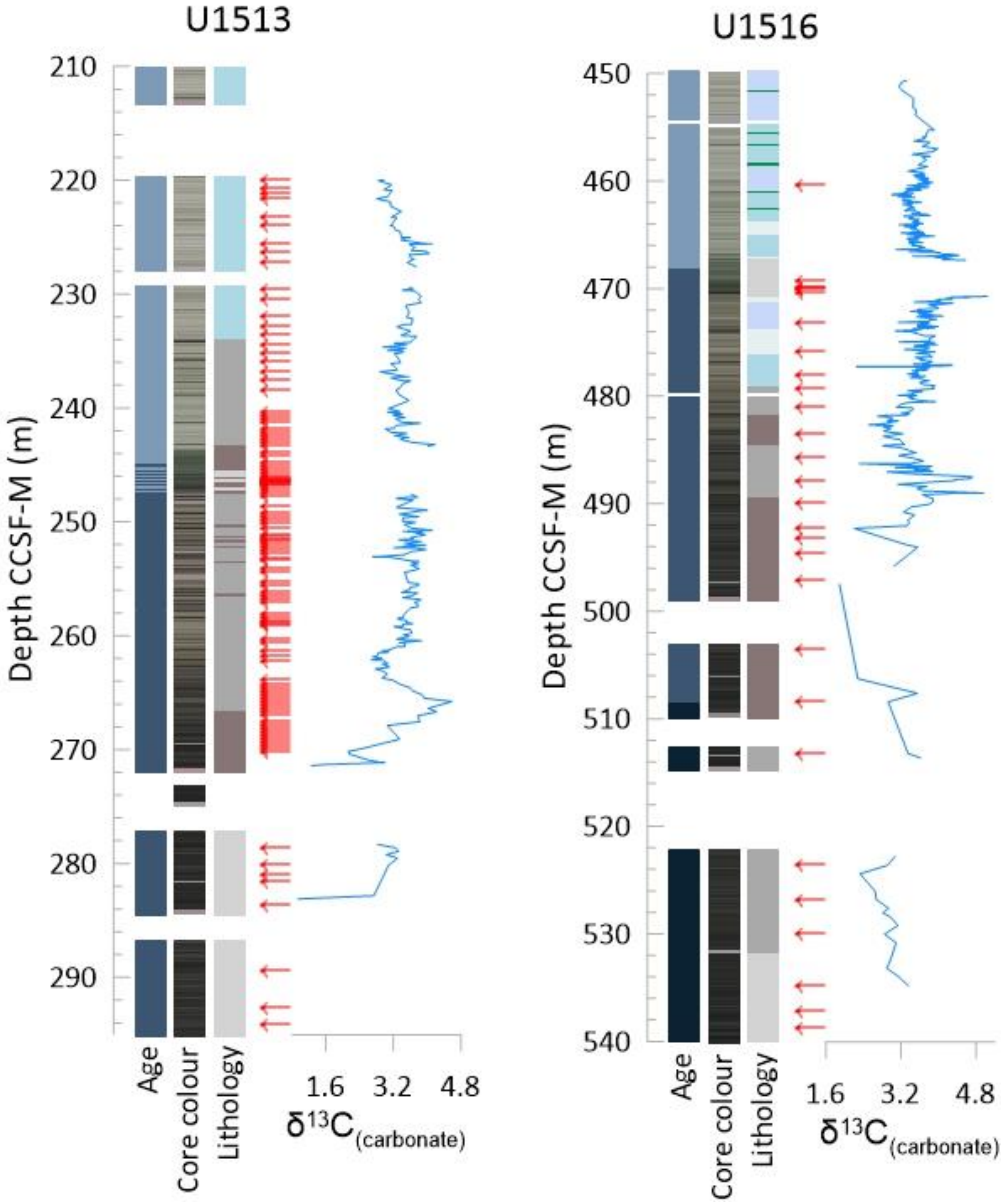

Absence of isorenieratine. Red arrows indicate sampled horizons tested for isorenieratene. No isorenieratane was found in any sample, indicating no photic zone euxinia across Ocean Anoxic Event 2 or the Mid-Cenomanian Event. Data are plotted next to sample age, core colour, lithology and the bulk $\delta^{13}C_{(carbonate)}$ data for reference.

Supplementary Figure 6 – Strong correlation between Neodymium and Strontium isotope values in fine fraction and bulk samples

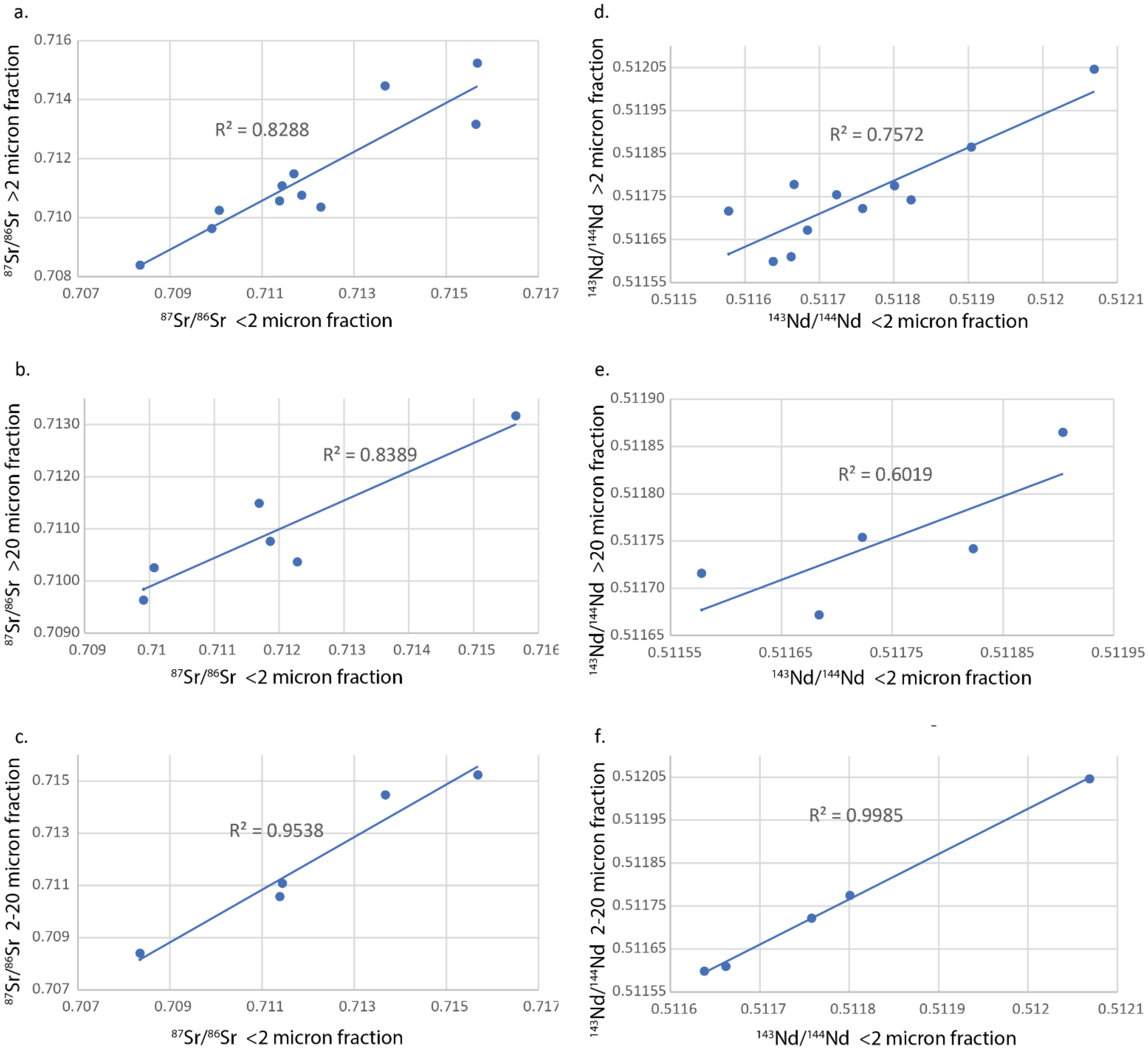

Strong correlation between Neodymium and Strontium isotope values in fine fraction and bulk samples. $^{87}Sr/^{86}Sr$ (a.-c.) show strong correlations between fine fraction (<2 micron), bulk, and 2-20 micron samples, where the linear trend strength is displayed in the R^2 value on each plot. a) Samples run with both fine fraction and all other fraction (>2 micron) sediments correlate strongly, where R^2 = 0.8288. This strong relationship is maintained when the 2-20 micron fraction is eliminated. b) shows bulk (>20 micron) against fine fractions samples, where R^2 = 0.8389. c) Fine fraction (<2 micron) and mid-size (2-20 micron) samples have a stronger correlation, where R^2 = 0.9538, though it should be noted that the sample size is only 5. Similar relationships are evidenced between the size fractions of εNd data (d. – f.). d) Fine fraction and all other fraction (>2 micron) size sediments have strongly correlating εNd data, where R^2 = 0.7572. A relatively strong relationship is maintained when the 2-20 micron fraction is eliminated. e) shows bulk (>20 micron) and fine fractions samples,

where $R^2 = 0.6019$. c) Fine fraction (<2 micron) and mid-size (2-20 micron) εNd samples demonstrate the strongest correlation, where $R^2 = 0.9985$, though, again, the sample size is only 5.

Supplementary Figure 7 – εNd values in southwest Australia

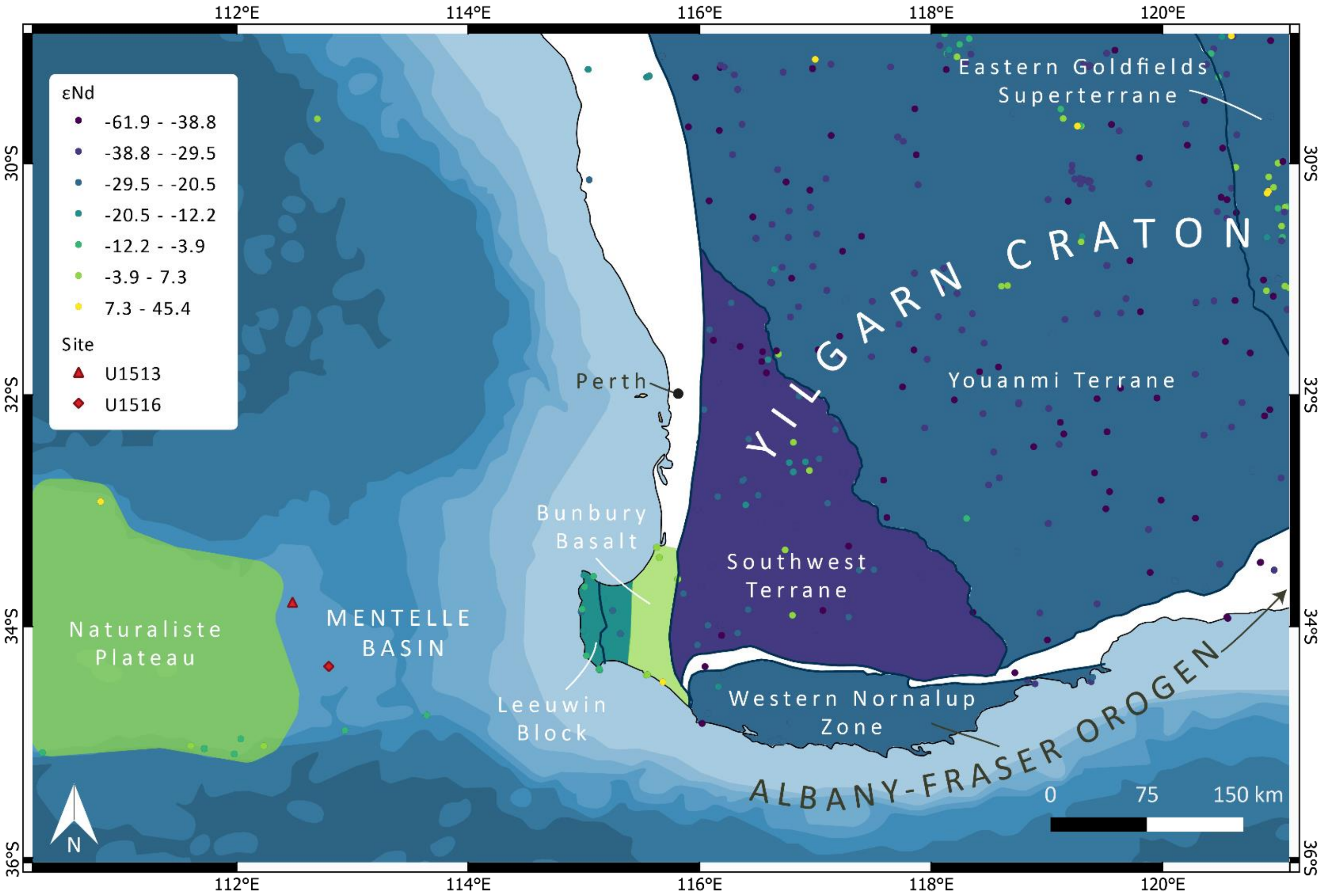

εNd isotope values in SW Australia. Each circular point represents a sediment or rock sample with assocaited εNd data, plotted in a colour representing the εNd value. Areas which may contribute sediments to the Mentelle Basin are labelled and coloured in according to the average εNd value of all samples within that area. Highly variable regions of geology, areas with few samples, and sedimentary basins for which εNd values are a mix of other source regions (such as the Perth Basin), are left white.

Supplementary Figure 8 - Sedimentation rate in the Mentelle Basin

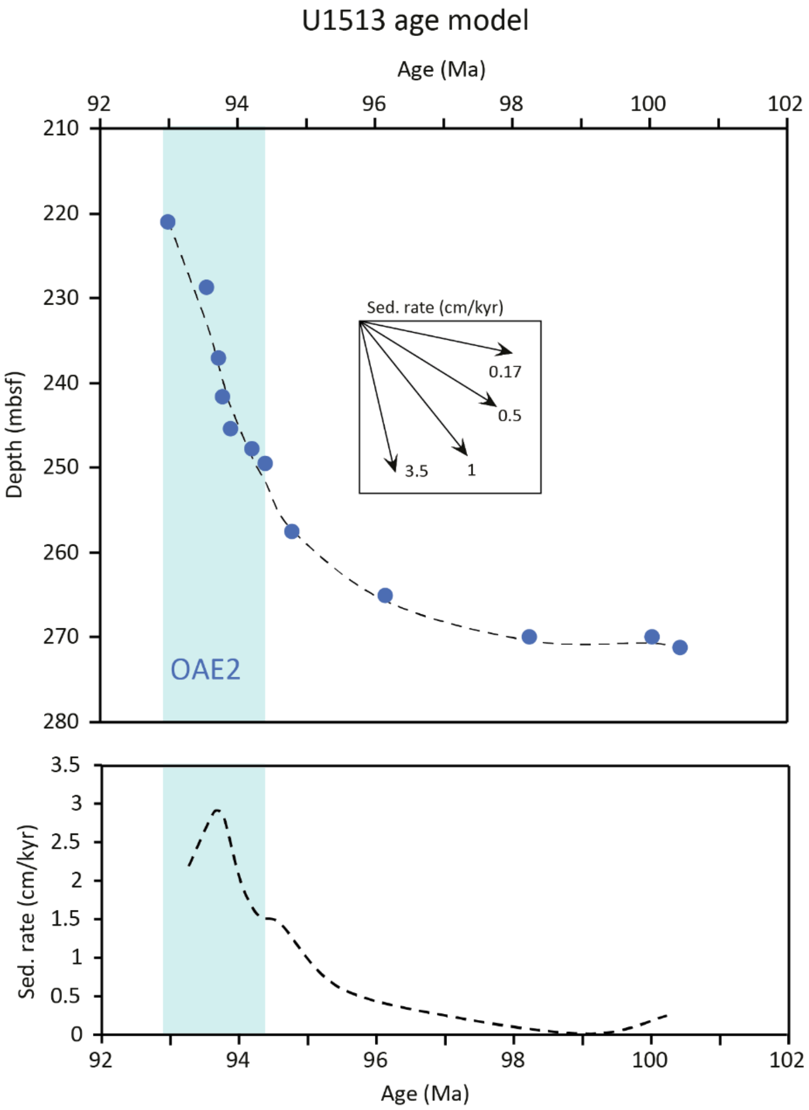

Sedimentation rate in the Mentelle Basin. Age depth plot above, and 1pt. smoothing spline demonstrating high sedimentation rate across Ocean Anoxic Event 2 below.

Supplementary Figure 9 –Strong correlation between mercury and Total Organic Carbon

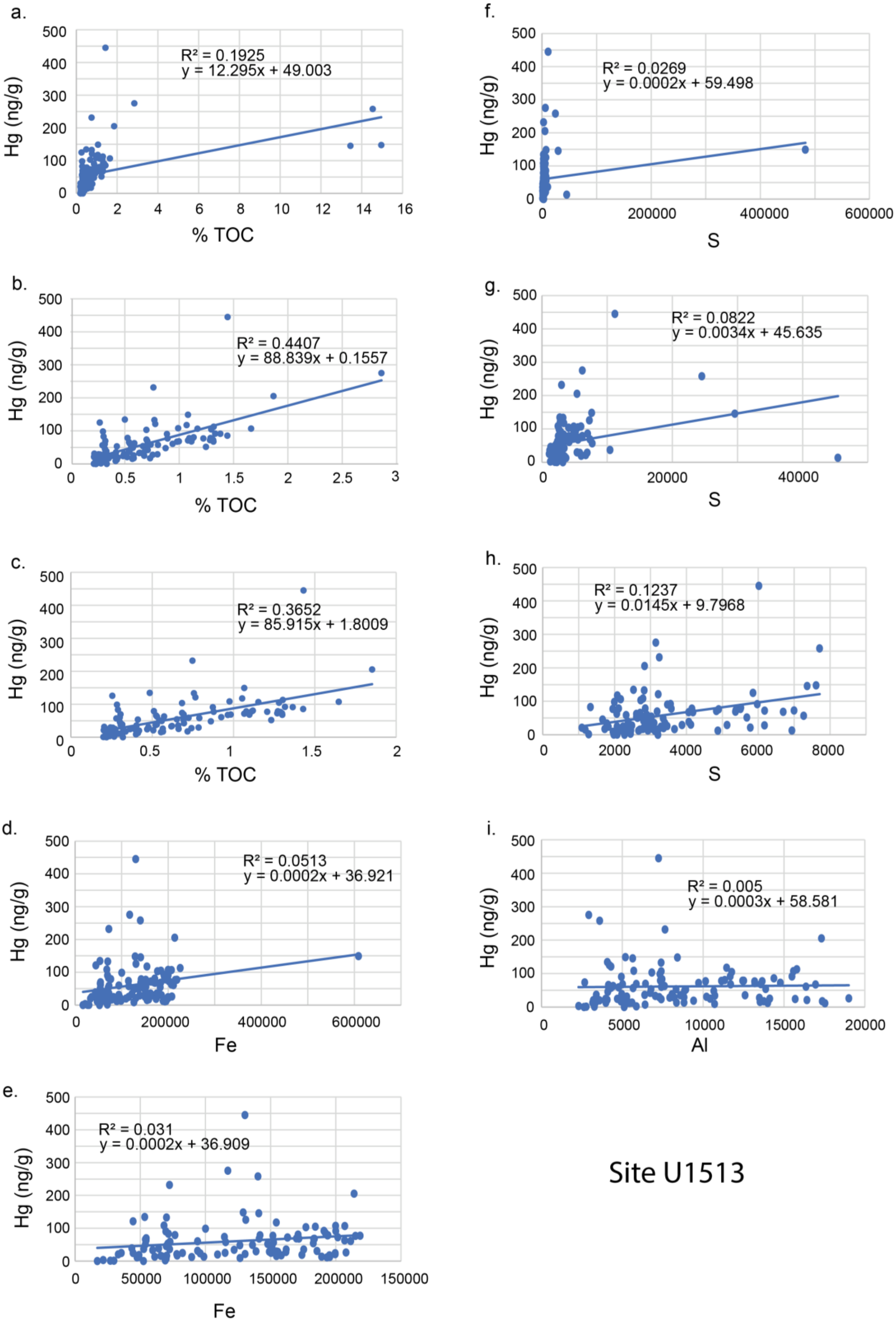

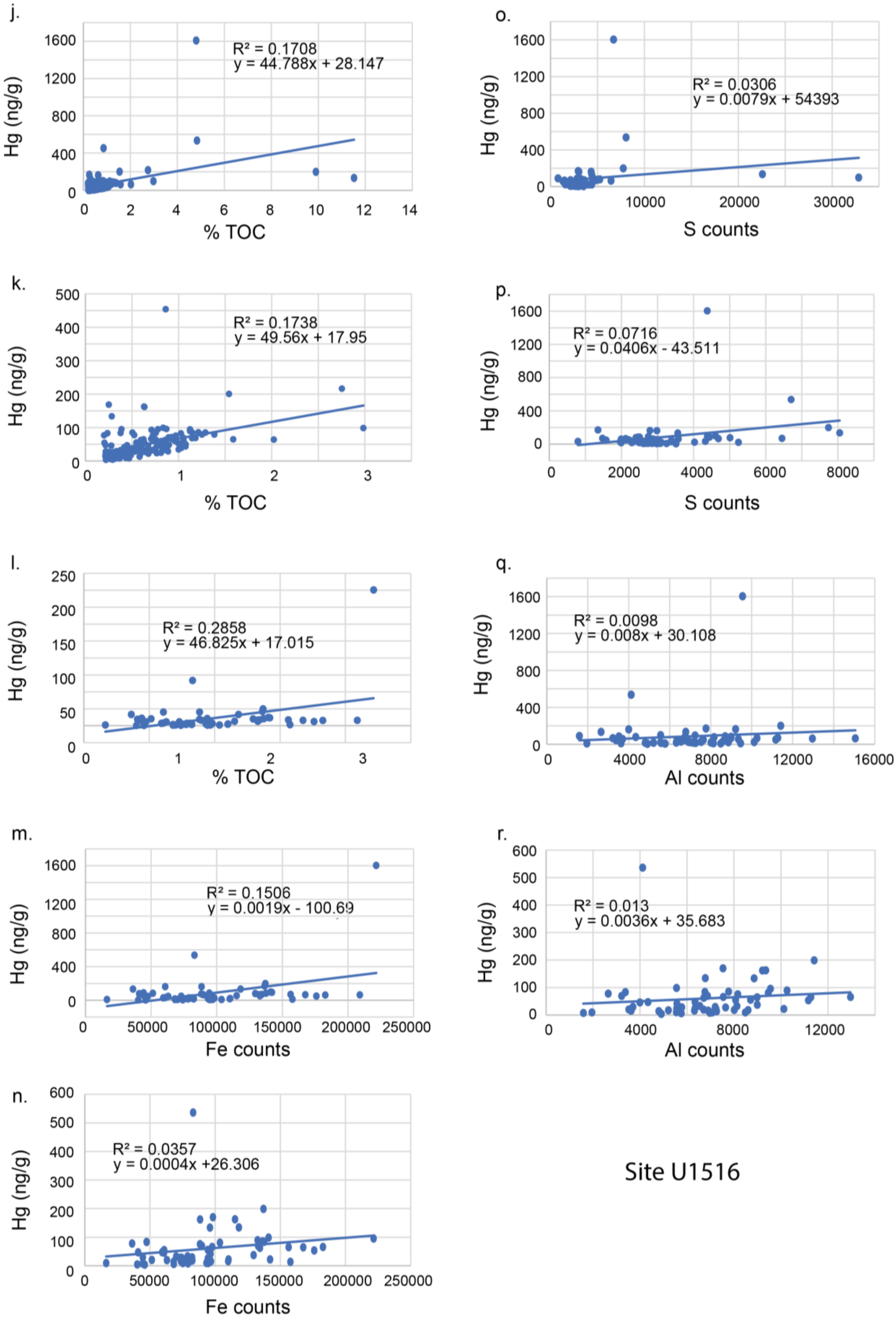

Strong correlation between mercury and Total Organic Carbon. Plots from U1513 (a – i) and U1516 (j – n) show relationship between Hg and TOC/Fe/Al/S. Linear trend strength is displayed in the R^2

value on each plot. Al, Fe and S data are counts from core scanning XRF. Relationships between Hg and Al/Fe/S are considerably weaker than the relationship between Hg and TOC, even after the removal of exceptionally high Fe or S values.

U1513 (a – i): a) TOC > 0.2%, R^2 = 0.1925. Value is heavily influenced by anomalously high TOC in samples from the black shale horizon within OAE2. b) 0.2 % > TOC < 3 %, removing the high TOC values. R^2 = 0.4407. c) further reducing the included values to 0.2 % > TOC < 2 % weakens the relationship between Hg and TOC - R^2 = 0.3652. e) Weak correlation between Hg and Fe counts - R^2 = 0.0513. f) correlation remains weak after excluding the sample with an exceptionally high Fe count (Fe < 150,000) - R^2 = 0.0310. f) Weak correlation between Hg and S counts - R^2 = 0.0269. g) Excluding the anomalously high S value (S < 50,000) still results in a weak correlation - R^2 = 0.0822. h) Excluding S counts > 8000 strengthens the correlation (R^2 = 0.1237), but this is still considerably weaker than the correlation between Hg and TOC. i) Weak correlation between Hg and Al - R^2 = 0.005.

U1516 (j – n): j) TOC > 0.2%, R^2 = 0.1708. Value influenced by anomalously high TOC in samples from the black shale horizons within OAE2. k) 0.2 % > TOC < 3 %, removing the high TOC values. R^2 = 0.1738. l) further reducing the included values to 0.2 % > TOC < 2 % strengthens the relationship between Hg and TOC - R^2 = 0.2858. m) Weak correlation between Hg and Fe counts - R^2 = 0.1506. n) correlation between Hg and Fe remains weak after excluding the sample with an exceptionally high Hg concentration (Hg <600) - R^2 = 0.0357. o) Weak correlation between Hg and S counts - R^2 = 0.0306. p) Excluding the anomalously high S value (S < 9,000) still results in a weak correlation - R^2 = 0.0716. q) Weak correlation between Hg and Al - R^2 = 0.0098. r) correlation between Hg and Al remains weak after excluding the sample with an exceptionally high Hg concentration (Hg <600) - R^2 = 0.0130.

Supplementary Figure 10 – Foraminifera SEM images

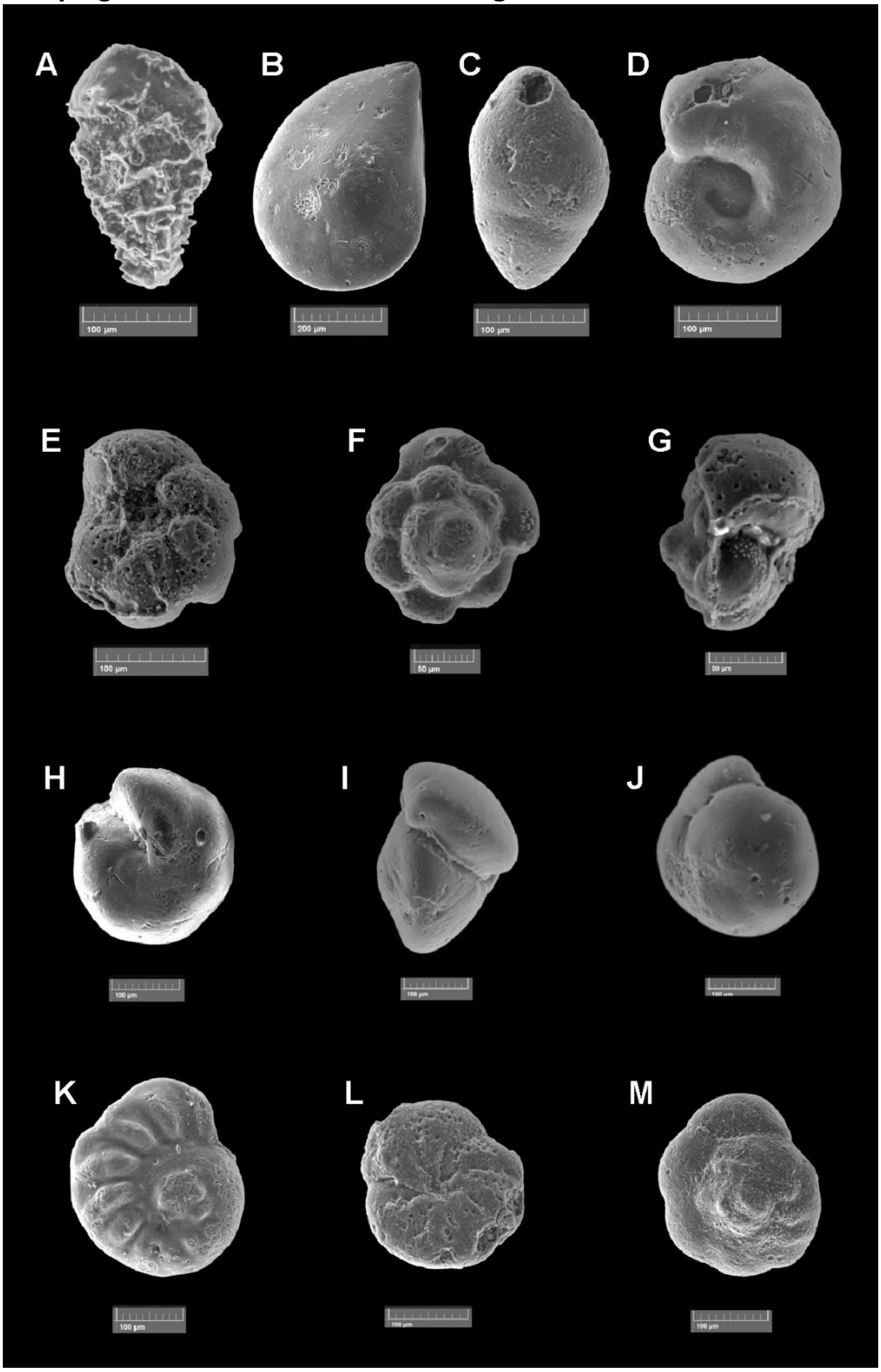

Foraminifera SEM images. A - *Tappanina laciniosa* (U1516D-4R-5W-0-3); **B** - *Lenticulina spp.* (U1516D-5R-3W-125-128); **C** - *Praebulimina nannina* (U1516D-3R-3W-52-55); **D** - *Gavelinella cenomanica* (U1516C-34R-2W-114-117); **E-G** - *Gyroidinoides quadratus* (U1516C-34R-3W-6-9); **H-J** - *Gyroidinoides lenticulus* (U1516C-34R-3W-6-9); **K** - *Gavelinella intermedia* (U1516C-34R-2W-114-117); **L-M** - *Osangularia utaturensis* (U1516D-3R-3W-52-55).

Supplementary Figure 11. Benthic foraminiferal stable isotope data

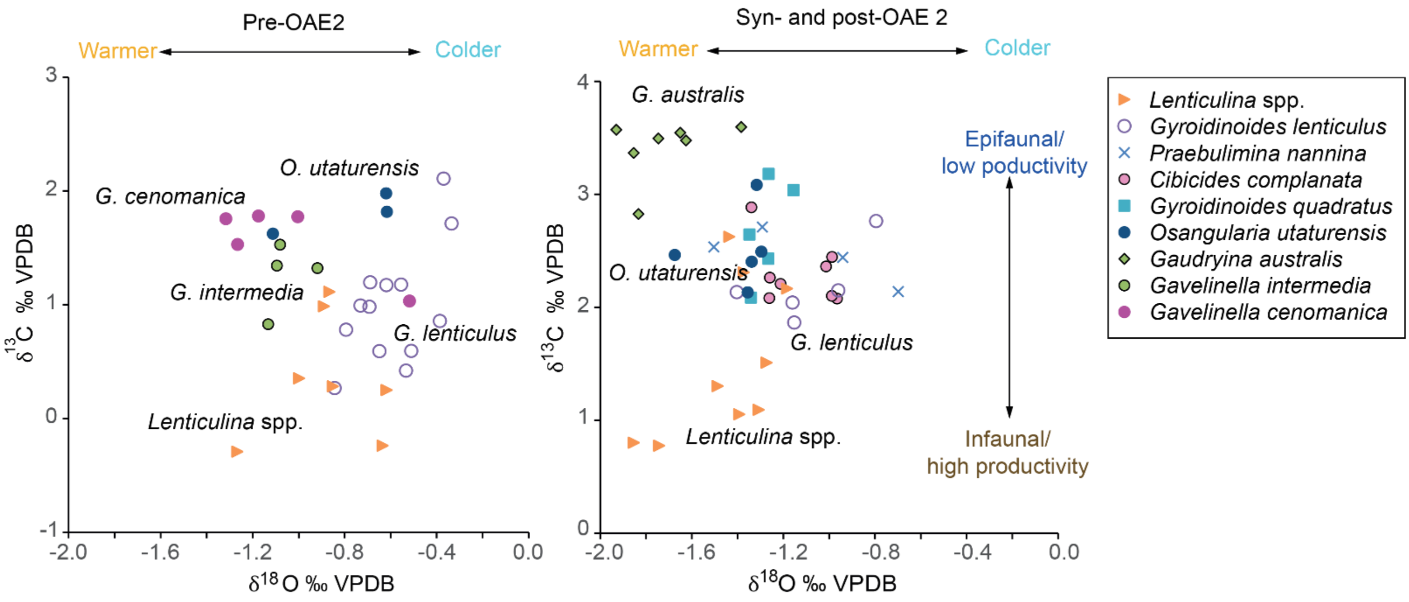

Benthic foraminiferal stable isotope data – $\delta^{13}C$ and $\delta^{18}O$ cross plots showing a shift in the benthic foraminiferal carbonate composition before and during/after Ocean Anoxic Event 2 (OAE2). Of the survivor species found before and after OAE2, the change in $\delta^{18}O$ (approximately - 1‰) is indicative of warmer temperatures, and of $\delta^{13}C$ (approximately +1‰) indicative of the global $\delta^{13}C$ shift of OAE2. The difference between species in both plots is indicative of species-specific offsets often related to sediment living depth e.g., lower $\delta^{13}C$ in deep sediment pore water profiles.

Supplementary Figure 12 – Proportion of infaunal species correlates with CA axis 1

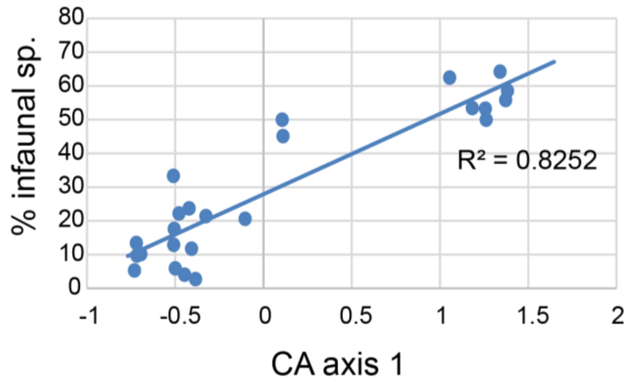

Proportion of infaunal species correlates with CA axis 1 - Correspondence analysis axis 1 plotted against the percentage of infaunal species in the assemblage at given sample depths. A strong relationship is seen between these variables, indicating that a component of change across OAE 2 may be attributed to the changing proportion of infaunal species of benthic foraminifera.

Supplementary Figure 13 – Calcium carbonate

a.

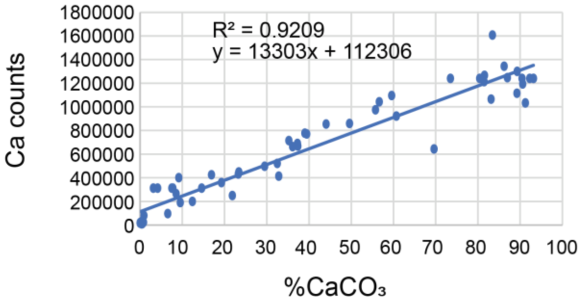

b.

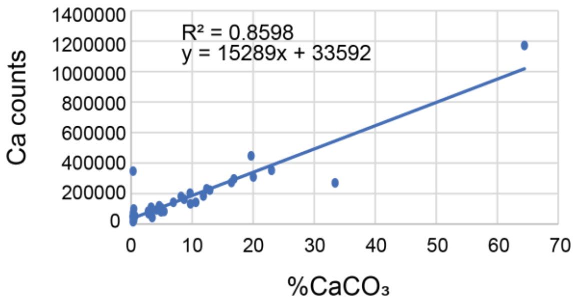

Calcium carbonate - % $CaCO_3$ plotted against Ca counts form shipboard XRF data at sites U1513 (a.) and U1516 (b.). The high R^2 value demonstrates a strong correlation. The linear equation is used to produce a secondary axis on figures, which translates Ca counts into an approximation of % $CaCO_3$.

Supplementary Material References

1. Frieling, J. *et al.* Effects of redox variability and early diagenesis on marine sedimentary Hg records. *Geochim. Cosmochim. Acta* **351**, 78–95 (2023).
2. Percival, L. M. E. *et al.* Does large igneous province volcanism always perturb the mercury cycle? Comparing the records of Oceanic Anoxic Event 2 and the end-Cretaceous to other Mesozoic events. *Am. J. Sci.* **318**, 799–860 (2018).
3. Huber, B. T., Hobbs, R. W., Bogus, K. A. & and the Expedition 369 Scientists. Proceedings of the International Ocean Discovery Program Volume 369 Australia Cretaceous Climate and Tectonics. *Proc. Int. Ocean Discov. Progr.* **369**, (2019).
4. Amos, H. M. *et al.* Global biogeochemical implications of mercury discharges from rivers and sediment burial. *Environ. Sci. Technol.* **48**, 9514–9522 (2014).
5. Petrizzo, M. R. *et al.* Exploring the paleoceanographic changes registered by planktonic foraminifera across the Cenomanian-Turonian boundary interval and Oceanic Anoxic Event 2 at southern high latitudes in the Mentelle Basin (SE Indian Ocean). *Glob. Planet. Change* **206**, 103595 (2021).
6. Petrizzo, M. R., MacLeod, K. G., Watkins, D. K., Wolfgring, E. & Huber, B. T. Late Cretaceous Paleoceanographic Evolution and the Onset of Cooling in the Santonian at Southern High Latitudes (IODP Site U1513, SE Indian Ocean). *Paleoceanogr. Paleoclimatology* **37**, (2022).
7. Petrizzo, M. R. *et al.* Biotic and Paleoceanographic Changes Across the Late Cretaceous Oceanic Anoxic Event 2 in the Southern High Latitudes (IODP Sites U1513 and U1516, SE Indian Ocean). *Paleoceanogr. Paleoclimatology* **37**, e2022PA004474 (2022).
8. Gradstein, F. M., Ogg, J. G., Schmitz, M. D. & Ogg, G. M. *The Geologic time scale, 2012*. (Elsevier Science, 2012).
9. Friedrich, O. Benthic foraminifera and their role to decipher paleoenvironment during mid-Cretaceous Oceanic Anoxic Events–the "anoxic benthic foraminifera" paradox. *Rev. micropaléontologie* **53**, 175–192 (2010).
10. Koutsoukos, E. A. M. & Hart, M. B. Cretaceous foraminiferal morphogroup distribution patterns, palaeocommunities and trophic structures: a case study from the Sergipe Basin, Brazil. *Earth Environ. Sci. Trans. R. Soc. Edinburgh* **81**, 221–246 (1990).
11. Koutsoukos, E. A. M., Leary, P. N. & Hart, M. B. Latest Cenomanian—earliest Turonian low-oxygen tolerant benthonic foraminifera: a case-study from the Sergipe basin (NE Brazil) and the western Anglo-Paris basin (southern England). *Palaeogeogr. Palaeoclimatol. Palaeoecol.* **77**, 145–177 (1990).
12. Bernhard, J. M. Characteristic assemblages and morphologies of benthic foraminifera from anoxic, organic-rich deposits; Jurassic through Holocene. *J. Foraminifer. Res.* **16**, 207–215 (1986).
13. Wendler, I., Huber, B. T., MacLeod, K. G. & Wendler, J. E. Stable oxygen and carbon isotope systematics of exquisitely preserved Turonian foraminifera from Tanzania—Understanding isotopic signatures in fossils. *Mar. Micropaleontol.* **102**, 1–33 (2013).
14. Gebhardt, H., Kuhnt, W. & Holbourn, A. Foraminiferal response to sea level change, organic flux and oxygen deficiency in the Cenomanian of the Tarfaya Basin, southern Morocco. *Mar. Micropaleontol.* **53**, 133–157 (2004).
15. Gertsch, B. *et al.* Middle and late Cenomanian oceanic anoxic events in shallow and

deeper shelf environments of western Morocco. *Sedimentology* (2010)
16. de España, N. & Gräfe, K. U. Late Cretaceous benthic foraminifers from the Basque-Cantabrian basin, northern Spain. *J. Iber. Geol.* **31**, 277–298 (2005).
17. Thomas, E. Late Cretaceous through Neogene deep-sea benthic foraminifera (Maud Rise, Weddell Sea, Antarctica). *Initial Reports Deep Sea Drill. Proj.* **113B**, 571–594 (1990).
18. Friedrich, O., Erbacher, J. & Mutterlose, J. Paleoenvironmental changes across the Cenomanian/Turonian boundary event (oceanic anoxic event 2) as indicated by benthic foraminifera from the Demerara Rise (ODP Leg 207). *Rev. micropaléontologie* **49**, 121–139 (2006).
19. Frenzel, P. Die benthischen Foraminiferen der Rügener Schreibkreide (Unter-Maastricht, NE-Deutschland). *Neues Jahrb. fur Geol. und Palaontologie - Abhandlungen* **3**, 1–361 (2000).
20. Alegret, L. & Thomas, E. Benthic foraminifera across the Cretaceous/Paleogene boundary in the Southern Ocean (ODP Site 690): Diversity, food and carbonate saturation. *Mar. Micropaleontol.* **105**, 40–51 (2013).
21. Basov, I. A. & Krasheninnikov, V. A. Benthic foraminifers in Mesozoic and Cenozoic sediments of the southwestern Atlantic as an indicator of paleoenvironment, Deep-Sea Drilling Project Leg-71. *Initial Reports Deep Sea Drill. Proj.* **71**, 739–787 (1983).
22. Holbourn, A., Henderson, A. S., MacLeod, N. & MacLeod, N. *Atlas of benthic foraminifera*. vol. 654 (Wiley Online Library, 2013).
23. Campbell, R. J. Calcareous nannofossil and foraminiferal analysis of the middle to upper cretaceous Bathurst Island Group, Northern Bonaparte Basin and Darwin Shelf, Northern Australia. (University of Western Australia Perth, 2003).
24. Nyong, E. E. & Olsson, R. K. A paleoslope model of Campanian to Lower Maestrichtian foraminifera in the North American basin and adjacent continental margin. *Mar. Micropaleontol.* **8**, 437–477 (1984).
