# Supplementary material for: Oceanic Anoxic Event 2 triggered by Kerguelen volcanism
Source: Nat Commun. 2024 Jun 15;15:5124. doi: 10.1038/s41467-024-49032-3 (PMC11180104; doi:10.1038/s41467-024-49032-3)
Supplement: Supplementary file 6 — Reporting Summary [file 41467_2024_49032_MOESM6_ESM.pdf]

nature portfolio

Corresponding author(s): Chloe Walker-Trivett

Last updated by author(s): Apr 26, 2024

Reporting Summary

Nature Portfolio wishes to improve the reproducibility of the work that we publish. This form provides structure for consistency and transparency in reporting. For further information on Nature Portfolio policies, see our Editorial Policies and the Editorial Policy Checklist.

Statistics

For all statistical analyses, confirm that the following items are present in the figure legend, table legend, main text, or Methods section.

n/a	Confirmed	
☐	☒	The exact sample size (*n*) for each experimental group/condition, given as a discrete number and unit of measurement
☐	☒	A statement on whether measurements were taken from distinct samples or whether the same sample was measured repeatedly
☒	☐	The statistical test(s) used AND whether they are one- or two-sided<br>*Only common tests should be described solely by name; describe more complex techniques in the Methods section.*
☐	☒	A description of all covariates tested
☐	☒	A description of any assumptions or corrections, such as tests of normality and adjustment for multiple comparisons
☐	☒	A full description of the statistical parameters including central tendency (e.g. means) or other basic estimates (e.g. regression coefficient) AND variation (e.g. standard deviation) or associated estimates of uncertainty (e.g. confidence intervals)
☒	☐	For null hypothesis testing, the test statistic (e.g. *F*, *t*, *r*) with confidence intervals, effect sizes, degrees of freedom and *P* value noted<br>*Give P values as exact values whenever suitable.*
☒	☐	For Bayesian analysis, information on the choice of priors and Markov chain Monte Carlo settings
☒	☐	For hierarchical and complex designs, identification of the appropriate level for tests and full reporting of outcomes
☒	☐	Estimates of effect sizes (e.g. Cohen's *d*, Pearson's *r*), indicating how they were calculated

Our web collection on statistics for biologists contains articles on many of the points above.

Software and code

Policy information about availability of computer code

Data collection	No software was used
Data analysis	PAST software version 2.17 C, GPlates 2.3.0 (Geological Information Model 1.6.0339), QGIS 3.28 (Firenze), Excel (Microsoft 365)

For manuscripts utilizing custom algorithms or software that are central to the research but not yet described in published literature, software must be made available to editors and reviewers. We strongly encourage code deposition in a community repository (e.g. GitHub). See the Nature Portfolio guidelines for submitting code & software for further information.

Data

Policy information about availability of data

All manuscripts must include a data availability statement. This statement should provide the following information, where applicable:

- Accession codes, unique identifiers, or web links for publicly available datasets
- A description of any restrictions on data availability
- For clinical datasets or third party data, please ensure that the statement adheres to our policy

The data that support the findings of this study can be found in the supplementary datasets, and are openly available in "figshare" at http://doi.org/10.6084/m9.figshare.25106123. Benthic foraminifera are curated at the Camborne School of Mines, University of Exeter, Penryn Campus, Cornwall TR10 9EZ. The DOI will become active upon publication.

Research involving human participants, their data, or biological material

Policy information about studies with human participants or human data. See also policy information about sex, gender (identity/presentation), and sexual orientation and race, ethnicity and racism.

Reporting on sex and gender	NA
Reporting on race, ethnicity, or other socially relevant groupings	NA
Population characteristics	NA
Recruitment	NA
Ethics oversight	NA

Note that full information on the approval of the study protocol must also be provided in the manuscript.

Field-specific reporting

Please select the one below that is the best fit for your research. If you are not sure, read the appropriate sections before making your selection.

☐ Life sciences ☐ Behavioural & social sciences ☒ Ecological, evolutionary & environmental sciences

For a reference copy of the document with all sections, see nature.com/documents/nr-reporting-summary-flat.pdf

Ecological, evolutionary & environmental sciences study design

All studies must disclose on these points even when the disclosure is negative.

Study description	Geochemical (stable C and O isotope, Hg concentration, Nd and Sr isotope data) and palaeontological analyses (benthic foraminiferal assemblages and isotopes) of marine sediment cores, to understand palaeoceanographic, volcanic, environmental, and biotic change through mid-Cretaceous Ocean Anoxic Events.
Research sample	International Ocean Discovery Program (IODP) marine sediment cores from the Mentelle Basin, SW Australia. Collected in November 2017 on Expedition 369. Samples selected as they span a relatively unbroken record across the time frame of interest, covering several ocean anoxic events, and are from a high palaeolatitude from the southern hemisphere, which is relatively understudied.
Sampling strategy	Samples were selected at regular intervals through the core, to provide a continuous record moving back through time through the mid-Cretaceous, which is when the events of interest occured. Sampling density of the core varies over depth to account for changes in sedimentation rate, so that various Ocean Anoxic Events are captured with a resolution comparable or higher than existing studies. Sample size was also determined by the quantity of sediment required for individual analyses (those that needed high volume of sediment are lower resolution, for example), to ensure sufficient material was available for all required analyses.
Data collection	Sediment cores were collected during IODP Expedition 369. C and O isotope data were collected by CAWT and JL. Hg and TOC data were recorded by CAWT. Nd and Sr isotope data were recorded by IM, DW and CAWT. Benthic foraminiferal data were recorded by TE.
Timing and spatial scale	Samples span ~122 - 90 Ma, taken from two sites in the Mentelle Basin, approximately 40km apart - U1513 and U1516. Data were collected between October 2018 and February 2023. Samples were kept refrigerated, or freeze-dried, for storage between analyses. Data collection occurred according to equipment availability.
Data exclusions	No data were excluded from analyses.
Reproducibility	For all organic geochemistry, both laboratory standards and sample repeats were carried out with each sample run, to ensure reproducibility. These repeats have been used to calculate standard deviation / error bands for instrument runs, which are published in the manuscript and in figures. Repeats were successful.
Randomization	Samples were disaggregated and homogenised, with each individual sample used for all analyses - with the exception of the samples used for benthic foraminifera - to ensure direct comparisons between sample horizons, therefore random allocation to experimental groups was not required. Disaggregation would have destroyed benthic foraminifera samples, so these came from adjacent horizons to those used for other analyses.
Blinding	Blinding not applicable for the collection or analysis of these palaeoecological/ geochemical data.

Did the study involve field work? ☐ Yes ☒ No

Reporting for specific materials, systems and methods

We require information from authors about some types of materials, experimental systems and methods used in many studies. Here, indicate whether each material, system or method listed is relevant to your study. If you are not sure if a list item applies to your research, read the appropriate section before selecting a response.

Materials & experimental systems

n/a	Involved in the study
☒	☐ Antibodies
☒	☐ Eukaryotic cell lines
☐	☒ Palaeontology and archaeology
☒	☐ Animals and other organisms
☒	☐ Clinical data
☒	☐ Dual use research of concern
☒	☐ Plants

Methods

n/a	Involved in the study
☒	☐ ChIP-seq
☒	☐ Flow cytometry
☒	☐ MRI-based neuroimaging

Palaeontology and Archaeology

Specimen provenance	Benthic foraminifera taken from sediment cores collected during International Ocean Discovery Program (IODP), Expedition 369 to the Mentelle Basin, SW Australia. Permit for study of micropalaeontological specimens from marine sediment cores not required.
Specimen deposition	Benthic foraminifera are stored in the Camborne School of Mines, University of Exeter, Tremough Campus, Cornwall
Dating methods	No new dates are provided.

☒ Tick this box to confirm that the raw and calibrated dates are available in the paper or in Supplementary Information.

Ethics oversight	No ethical guidance or approval was required - samples are from IODP, College Station, Texas, collected in line with standard IODP policies.

Note that full information on the approval of the study protocol must also be provided in the manuscript.

Plants

Seed stocks	NA
Novel plant genotypes	NA
Authentication	NA
